# Targeted Interventions for Improved Equity in Maternal and Child Health in Low- and Middle-Income Settings: A Systematic Review and Meta-Analysis

**DOI:** 10.1371/journal.pone.0066453

**Published:** 2013-06-20

**Authors:** Mats Målqvist, Beibei Yuan, Nadja Trygg, Katarina Selling, Sarah Thomsen

**Affiliations:** 1 International Maternal and Child Health, Department of Women’s and Children’s Health, Uppsala University, Uppsala, Sweden; 2 Peking University, China Center for Health Development Studies, Beijing, China; 3 Department of Public Health Sciences, Global Health/IHCAR, Karolinska Institutet, Solna, Sweden; The University of Adelaide, Australia

## Abstract

**Background:**

Targeted interventions to improve maternal and child health is suggested as a feasible and sometimes even necessary strategy to reduce inequity. The objective of this systematic review was to gather the evidence of the effectiveness of targeted interventions to improve equity in MDG 4 and 5 outcomes.

**Methods and Findings:**

We identified primary studies in all languages by searching nine health and social databases, including grey literature and dissertations. Studies evaluating the effect of an intervention tailored to address a structural determinant of inequity in maternal and child health were included. Thus general interventions targeting disadvantaged populations were excluded. Outcome measures were limited to indicators proposed for Millennium Development Goals 4 and 5. We identified 18 articles, whereof 15 evaluated various incentive programs, two evaluated a targeted policy intervention, and only one study evaluated an intervention addressing a cultural custom. Meta-analyses of the effectiveness of incentives programs showed a pooled effect size of RR 1.66 (95% CI 1.43–1.93) for antenatal care attendance (four studies with 2,476 participants) and RR 2.37 (95% CI 1.38–4.07) for health facility delivery (five studies with 25,625 participants). Meta-analyses were not performed for any of the other outcomes due to scarcity of studies.

**Conclusions:**

The targeted interventions aiming to improve maternal and child health are mainly limited to addressing economic disparities through various incentive schemes like conditional cash transfers and voucher schemes. This is a feasible strategy to reduce inequity based on income. More innovative action-oriented research is needed to speed up progress in maternal and child survival among the most disadvantaged populations through interventions targeting the underlying structural determinants of inequity.

## Background

The end date of the Millennium Development Goals (MDG) –2015– is approaching fast and evidence shows that the target levels of under-five and infant mortality (MDG4) and maternal mortality (MDG5) are not going to be met at a global level [1], [2]. However, some countries are making good progress, particularly on MDG4, and have already reached the reductions strived for, especially in Latin America and Southeast Asia [2]. Countries like Vietnam and Brazil have managed to increase child survival over the past decade, primarily due to a rapid economic transition and improved standards of living in the general population [2]. But amidst these successes more and more evidence points to unequal distribution of the newly acquired wealth, with certain population groups being left behind. These disadvantaged populations present negative health outcomes at a stagnant high level, with maternal and child mortality far above the average [3]. Thus it has become clear that general economic development is not enough for improving health for all, but that policy makers and health care planners also need to take the health of disadvantaged groups into consideration to ensure sustainable development.

In order to reduce inequities in health there are two main approaches that can be applied, either to strive for universal coverage of health care interventions, with a special focus on the most vulnerable groups, or to apply targeted interventions directed at marginalised population groups [4]. Universal coverage of health care is a prerequisite for an equitable health system, but to override the structural drivers of inequity and ensure equal opportunity, policies need to be modelled to proactively promote health for the disadvantaged with a clear understanding of the mechanisms causing inequity. Thus universal interventions like free health care for all or an even geographical distribution of health facilities may need to be supplemented by targeted interventions focusing on special needs and obstacles to equitable care. Examples like affirmative action, anti-discrimination laws or support based on needs assessments play an important role in these efforts. Even though there are some systematic reviews evaluating the effects of targeted interventions such as conditional cash transfers [5], [6] and culturally adapted programs [7], [8] we have not found any systematic compilations of interventions specifically tailored to reduce inequity in maternal and child health in low- and middle income countries. The aim of the present systematic review is therefore to display the evidence of such targeted interventions in the field of maternal and child health and to compile the available evidence of the effectiveness of such interventions to reduce inequity.

## Methods

### Search Strategy

**Figure 1:**
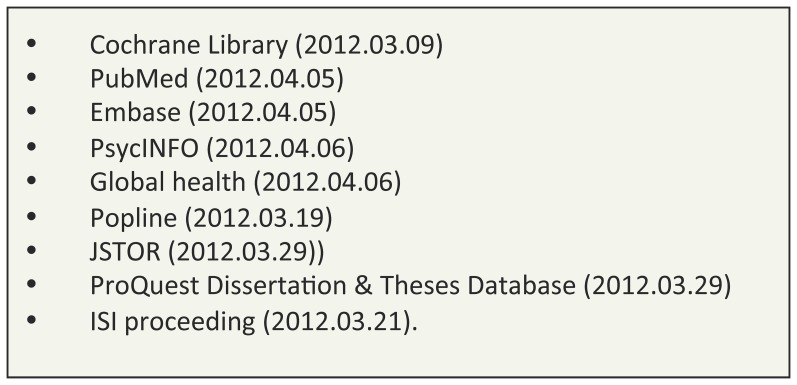
Databases utilized. Searches performed from inception dates to date noted.

We identified primary studies in all languages by searching nine databases about health and social science, including grey literature and dissertation databases (Figure 1). The terms about maternal or child health, equity or disadvantaged populations, developing countries and intervention studies were combined in the same search strategy in each database (Appendix S1). Citation tracking in relevant articles was performed to identify additional relevant papers. No preset study protocol was used or registered for this systematic review.

### Inclusion Criteria

**Figure 2:**
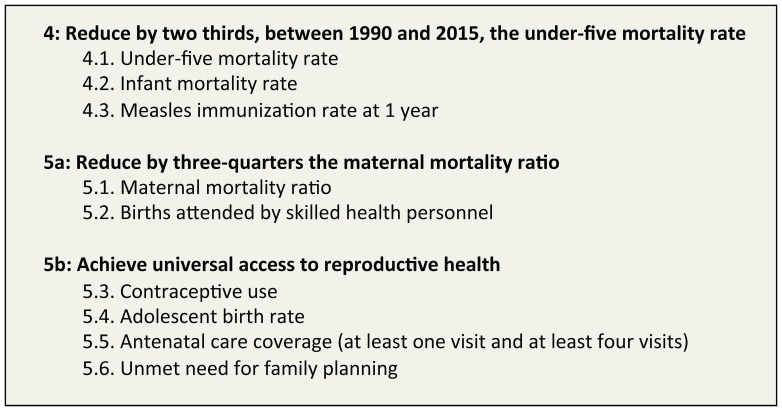
Targets and Indicators of MDGs 4 and 5.

Studies with experimental or observational study designs that assessed the effects of different interventions on maternal and child health were included, including randomized controlled trials, cluster randomized controlled trials, quasi-randomized controlled trials, controlled before-after studies, time series studies, before and after studies, cohort studies and case control studies. The category of countries included was based on the World Bank List of Economies 2011 identifying low and middle-income settings. In terms of outcome measures, we only considered studies reporting the effects of interventions on MDGs 4 and 5 indicators (Figure 2).

In the first step we included all interventions designed to improve maternal and/or child health in any segment of the population (both universal interventions and targeted interventions) and/or those interventions specifically designed to reduce inequities in maternal and/or child health in low and middle-income countries. For final inclusion only the interventions specifically designed to reduce inequities by targeting obstacles for health posed by structural determinants like poverty, ethnicity and education, for example subsidies for the poor, intervention addressing specific cultural customs or information directed towards the illiterate, were included. Thus, implementing a general intervention in a disadvantaged population or setting did not meet the inclusion criteria.

### Study Selection

Two review authors independently scanned titles and abstracts of all articles obtained from the search to exclude those studies that were not about effectiveness of interventions to improve maternal and child health. All remaining intervention studies were reviewed in full if needed and potential studies about effectiveness of interventions on disadvantaged populations or equity were kept. In the next step the full texts were obtained and screened in order to find studies evaluating effects of interventions fulfilling the final inclusion criteria, i.e. incorporating a strategy to target a structural determinant of health in a disadvantaged population. During selection process, all review authors resolved any disagreements on inclusion by discussion.

### Data Extraction and Quality Assessment

Key information from included full texts was extracted and the quality of each study was assessed by one review author and checked by a second. We assessed study quality using a six-item checklist of quality of execution adapted from the criteria developed for the Effective Public Health Practice Project in Hamilton, Ontario [9]. We extracted intervention content, target population, study design, and outcome data.

### Data Synthesis

We firstly categorized and summarized the characteristics of included studies, including intervention content, outcome indicators, and studies’ methodological quality. Studies were then categorized according to the character of the intervention. Combined with quality of studies, we analysed if the conclusion was influenced by studies’ methodological quality. Given the wide range of included studies a narrative synthesis of the result was undertaken.

### Data Analysis

Meta-analyses were conducted when appropriate and possible. Four meta-analyses were performed, two for each outcome depending on study design. Studies measuring rates before and after intervention were grouped as were studies displaying differences between intervention and control areas. In case only proportions were accounted for a sample size of 100 was assumed. Calculations were carried out in R [10], using the Metafor module [11], utilizing a random effect model. Results were displayed as pooled effect (RR) and forest plots.

## Results

**Figure 3:**
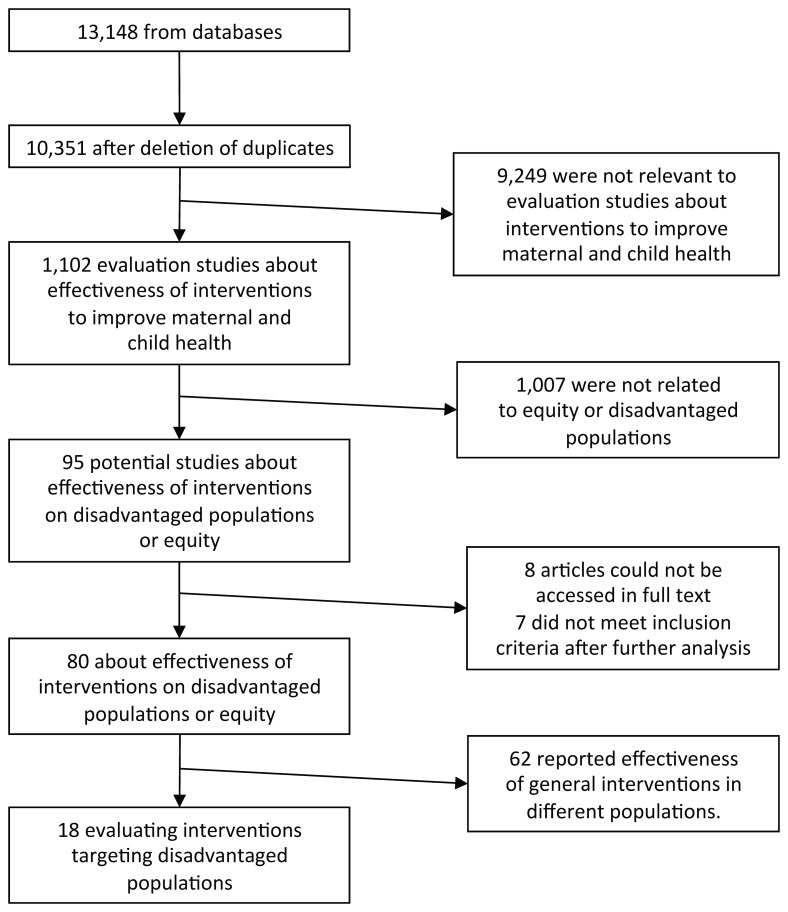
Flow-chart for study selection.

We found 13,148 references from 9 databases, and 10,351 were left after deleting duplicates, of which 1,101 were evaluation studies about interventions to improve maternal and children health. Of 1,101 references, only 94 were related the effectiveness of interventions on disadvantaged populations or different effects between different segments of populations. After reading the full texts of these potentially relevant studies, we finally included 18 articles evaluating interventions designed to target disadvantaged populations (Figure 3). Of these 18 studies, 15 evaluated interventions using some kind of incentive, material or cash, two (2) studies evaluated the effect of policy change on the health care utilization of disadvantaged groups and only one (1) study was found with an intervention targeting a cultural tradition with a negative influence on child survival (Table 1).

**Table 1:** Included studies.

Article (Author, title, publication)	Year	Context	Intervention design	Study design	Main results	MDG indicator	Quality rating
***Interventions with incentives; conditional-cash transfers, voucher schemes, material rewards***							
Agha S. Changes in the proportion of facility-based deliveries and related maternal health services among the poor in rural Jhang, Pakistan: Results from a demand-side financing intervention. International Journal for Equity in Health 2011;10∶57.	2011	Rural Pakistan	Vouchers distributed to pregnant women from poor households admitting free antenatal, delivery and postnatal care. Health providers reimbursed for received vouchers.	Repeated cross-sectional study comparing women from 10 intervention councils and 10 control councils.	Significant increases in institutional delivery rate among poor women in intervention area compared to non-poor and control area. Non-significant effect on ANC and PNC use and no effect on family planning	5.2; 5.5	Moderate
Agha, S. Impact of a maternal health voucher scheme on institutional delivery among low income women in Pakistan. Reprod Health 2011; 8∶10.	2011	Dera Ghazi Khan City, Pakistan	Vouchers distributed to pregnant women from poor households admitting free antenatal, delivery and postnatal care. Health providers reimbursed for received vouchers.	Cross-sectional study comparing randomly selected women who delivered before and during the intervention period.	The change in use of ANC and institutional delivery varied between different income groups. ANC use increased mostly in the middle quintiles, while institutional delivery rate increased among the poorest.	5.2; 5.5	Strong
Ahmed S. & M.M. Khan. Is demand-side financing equity enhancing? Lessons from a maternal health voucher scheme in Bangladesh. Soc Sci Med, 2011; 72(10): 1704–10.	2011	Rural Bangladesh	Vouchers distributed to all pregnant women in the study area admitting free antenatal, delivery and postnatal care. Health providers reimbursed for received vouchers.	Non randomized trial with one intervention and one control area	Voucher recipients were more likely to deliver at a health facility and to attend antenatal care compared to non-recipients. The largest effect of the voucher scheme was seen among the poor recipients.	5.2; 5.5	Moderate
Banerjee A.V. et al. Improving immunisation coverage in rural India: clustered randomised controlled evaluation of immunisation campaigns with and without incentives. BMJ 2010; 340:c2220.	2010	Rural Rajasthan, India	Immunization campaign with or without material incentives.	Clustered randomized controlled study	Small incentives have large positive impacts on the uptake of immunization services in resource poor areas.	4.3	Strong
Barham T. & J.A. Maluccio. Eradicating diseases: the effect of conditional cash transfers on vaccination coverage in rural Nicaragua. Journal of Health Economics 2009; 28∶611–21.	2009	Rural Nicaragua	Conditional cash transfer to mothers for health (vaccination a and nutrition) and education (workshop)	Clustered randomized controlled study	The program led to large increases in vaccination coverage with pronounced effects in hard-to reach populations.	4.3	Strong
Barham T. Impact of the Mexican conditional cash transfer program on immunization rates	2005	Mexico	Conditional cash transfers to families that conveyed to a pre-set health promotion program (Progresa)	Clustered randomized controlled study	An increase of three percentage points was seen in the intervention area compared to control area	4.3	Strong
Feldman B.S. et al. Contraceptive use, birth spacing, and autonomy: an analysis of the Oportunidades program in rural Mexico	2009	Mexico	Conditional cash transfer to poor rural pregnant women§	Repeated cross-sectional surveys in 1997, 1998, 2000 and 2003 in a cluster-randomized sample.	Beneficiaries of the program increased their use of modern contraceptives more than controls in the early stage of the program. Later on no difference between intervention and control could be detected.	5.3	Moderate
Ir P. et al. Using targeted vouchers and health equity funds to improve access to skilled birth attendants for poor women: A case study in three rural health districts in Cambodia. BMC Pregnancy and Childbirth 2010; 10∶1.	2010	Cambodia	Health equity fund (HEF), vouchers and supplier incentives (performance based contracting)	Cross-sectional study using data from the routine health information system.	Increase in facility delivery from 16.3% to 44.9% in intervention area	5.2	Weak
Lim S.S. et al. India's Janani Suraksha Yojana, a conditional cash transfer programme to increase births in health facilities: an impact evaluation. Lancet 2010; 375(9730): 2009–23.	2010	India	Vouchers distributed to pregnant women from poor households admitting free antenatal, delivery and postnatal care and a monetary reimbursement for mothers and health providers after completed services (Janani Suraksha Yojana program).	Cross-sectional study using data from nationwide district-level household surveys performed in 2002–04 and 2007–09.	Increases in ANC and facility delivery. However, the poorest and least educated women did not always have the highest odds of receiving cash payments.	5.2; 5.5	Moderate
Mavalankar, D. et al. Saving mothers and newborns through an innovative partnership with private sector obstetricians: Chiranjeevi scheme of Gujarat, India. Int J Gynaecol Obstet, 2009;107(3):271–6.	2009	Gujarat, India	Voucher scheme providing free obstetric care at private providers.	Cross-sectional study comparing reported data with estimated data.	Facility-based deliveries increased from 27% to 53%. Estimated improvements in maternal and neonatal mortality	5.1; 5.2	Weak
Meuwissen, L. E., Impact of accessible sexual and reproductive health care on poor and underserved adolescents in Managua, Nicaragua: a quasi-experimental intervention study. J Adolescent Health 2006; 38∶56.e1-56.e9.	2006	Disadvantaged areas in Managua, Nicaragua	Voucher scheme targeting male and female adolescents in disadvantaged areas.	Cross sectional comparison between intervention and control groups.	At schools, sexually active voucher receivers had a significantly higher use of modern contraceptives than non-receivers.	5.3	Moderate
Morris S. S. Monetary incentives in primary health care and effects on use and coverage of preventive health care interventions in rural Honduras: cluster randomised trial. Lancet 2004; 364∶2030–37.	2004	Rural Honduras	Monetary vouchers paid to women in households in beneficiary municipalities in addition to resources to local health teams combined with a community-based nutrition intervention.	Cluster randomized controlled trial comparing intervention and control area before and after intervention.	The household-level intervention had a large impact (15–20 percentage points; p<0·01) on the reported coverage of antenatal care. Measles immunisation rate was not affected.	4.3; 5.5	Strong
Nguyen, H.T.H., et al., Encouraging maternal health service utilization: An evaluation of the Bangladesh voucher program. Social Science and Medicine, 2012; 74(7): 989–996.	2012	32 sub- districts in Bangladesh	Vouchers distributed to all pregnant women in the study area admitting free antenatal, delivery and postnatal care. Health providers reimbursed for received vouchers.	Cross sectional comparison between intervention and control groups using data from household survey in 2009.	Overall increased use of qualified providers for ANC, delivery and PNC with substantially larger program effects among the poorest women.	5.2,; 5.5	Moderate
Rob U., M. Rahman & B. Bellows. Using vouchers to increase access to maternal healthcare in Bangladesh. Int Q Community Health Educ 2009; 30(4):293–309.	2009	Bangladesh	Vouchers distributed to pregnant women from poor households admitting free antenatal, delivery and postnatal care. Health providers reimbursed for received vouchers.	Repeated cross-sectional before-after intervention.	Increased ANC, facility delivery and PNC	5.2; 5.5	Weak
Sosa-Rubi S.G. et al. Learning effect of a conditional cash transfer programme on poor rural women's selection of delivery care in Mexico. Health Policy Plan 2011; 26(6): 496–507.	2011	Mexico	Conditional cash transfer to poor rural pregnant women	Repeated cross-sectional surveys in 1998, 2003 and 2007.	A so called learning effect was found, illustrating how women with a longer exposure to the program had higher probability of their last delivery to be attended by a skilled personnel vs. Traditional midwife. The most disadvantaged women had however less access to skilled birth attendance.	5.2; 5.5	Moderate
***Policy interventions***							
McQuestion M. J. Evaluating program effects on institutional delivery in Peru. Health Policy 2006; 77∶221–232.	2006	Peru	SMI Program (provided delivery care coverage to Peru’s poorest households, 1998) and Proyecto 2000 (sought to improve the quality of EmOC and increase utilization of public EmOC facilities, 1996–2002)	Cross sectional comparison between intervention and control groups	A mother enrolled in the SMI Program was more likely to have delivered her last child in a public EmOC, controlling for household constraints. Residence in a Proyecto 2000 treatment area did not significantly affect the choice. A cross-level interaction term was insignificant, indicating the two program effects were independent.	5.2	Strong
Uddin M.J. et al. Improving low coverage of child immunization in rural hard-to-reach areas of Bangladesh: findings from a project using multiple interventions. Vaccine 2012; 30(2):168–79.	2012	Bangladesh	EPI program in combination with policy change to eliminate barriers relating to geographical boundaries.	Pre-post intervention surveys	Increased overall coverage of measles vaccine coverage but	4.3	Strong
***Culturally adapted interventions***							
Meegan, M. E. Effect on neonatal tetanus mortality after a culturally-based health promotion programme. Lancet 2001; 358∶640–41.	2001	Kenya	Locally recruited traditional birth attendants, whose responsibilities included peer group education, prenatal monitoring, delivery, postpartum follow-up, and referral where necessary, delivered health promotion. Traditional birth attendants were given individual packs for each birth.	Time-series using cross-sectional data.	After introduction of the programme, neonatal tetanus rates fell sharply, and by 1988 death rates had dropped to 0·75 (range 0–3) per 1000 births in the intervention areas compared to 82 (74–93) per 1000 in control areas. Death rates in intervention communities did not rise again between 1988 and 1999. Total mortality rates in children aged less than 6 weeks fell from 307 to 50 per 1000 in intervention areas, while they went from 233 to 294 in the control area over the same period.	4.2	Moderate

### Incentive Strategies for MDG5

All in all fifteen studies reported on effectiveness of interventions using incentives to improve health outcomes related to MDG 4 and 5 (Table 1). Eleven of the fifteen studies were rated as ‘moderate’ or ‘strong,’ according to the applied rating system. Providing incentives of some sort, monetary or material, is a way of targeting the structural determinant income/wealth. Poverty is a strong driver of inequity and by providing some kind of award for health care utilisation it is not only possible to increase motivation for care seeking but also to alleviate financial barriers to health care utilisation.

Maternal mortality (MDG 5.1) is a rare event even in settings with high MMR. There are considerable methodological difficulties associated with measuring maternal mortality and most studies therefore rely on estimations. In a study from Gujarat, India, Mavalankar and colleagues developed a partnership scheme with private obstetricians, contracting them to provide skilled care at delivery, including EmOC, to poor women without charge [12]. In return they would be compensated for services provided by the government of Gujarat through a voucher scheme (Chiranjevee). In total, 800 obstetricians were contracted and more than 269,000 poor women delivered in a private facility. By comparing the estimated maternal mortality rate within the study area with the reported rate this intervention scheme seems to have reduced maternal deaths considerably, from expected 588 deaths in 269,942 births to 156–208 (corrected for assumed under-reporting). The study quality was however weak due to study design and failing to control for relevant confounders.

Ten studies displayed the effect on institutional delivery rate (MDG 5.2) and antenatal care attendance (MDG 5.5) of different conditional cash transfer and voucher programs. All of these studies, whereof seven from south Asia (Bangladesh, India and Pakistan) [13]–[18], one from South-east Asia (Cambodia) [19] and two from Latin America (Mexico and Honduras) [20], [21], showed a positive impact on health facility delivery rate among the poorer segments of society. The effect on antenatal care attendance was however more ambiguous and some studies failed to show an impact of the applied intervention.

The intervention designs varied, applying different levels of reimbursements of patients and providers and mixed administrative routines. Two studies of moderate quality evaluated a voucher program in Bangladesh where all pregnant women in the study area were identified and given a set of vouchers that would render them antenatal care, delivery and postnatal care services for free [13], [14]. Health care providers were then compensated for each voucher they received. The studies showed a significant increase in the overall use of antenatal and delivery care services [14] and a stronger demand-increasing effect among the poor [13]. A third study from Bangladesh applied the same voucher scheme but chose to include only the poorest women [15]. This study also was able to demonstrate a substantial increase in antenatal care and facility delivery rate. The quality of the study was however weak due to study design and lack of confounder analysis.

Two studies of moderate quality from Pakistan, one in an urban and another in a rural setting, applied a similar voucher program as in the studies from Bangladesh except that the voucher booklets, which were valued at about 50 USD, had to be bought for around 1 USD [16], [17]. The vouchers were only sold to women identified as poor. Both studies showed an increase in antenatal care attendance and facility delivery rate in the fourth and fifth quintiles of around 20 percentage points.

In 2005, the government of India launched a country-wide conditional cash transfer scheme called *Janani Surkahsa Yojana* (JSY). This program not only provides free services to eligible women but also provides a monetary reward once the women have utilised maternity care. Furthermore, local health workers known as ASHAs (Accredited Social Health Activists) were mobilised to identify pregnant women and help them to get to a health facility. ASHAs were then economically compensated for each pregnant woman they brought to the health services, thus creating incentives at multiple points to strengthen the effect of the program. Despite this, the implementation of JSY was not consistent across states. However, a study by Lim et al of moderate quality demonstrated a significant increase in antenatal care and institutional delivery as a result of the program, even if the poorest and least educated women were not always the ones with the highest odds of receiving JSY payments [18]. The study by Lim et al was also able to show effects on child mortality through this incentive program. JSY payment was associated with a reduction of neonatal mortality of 2.3/1000 live births.

One study not situated in Asia evaluated the long-term effects of a cash transfer program, *Oportunidades*, in Mexico, with a slightly different intervention design compared to previous studies [20]. Instead of vouchers or other ways of providing services free of charge, the *Oportunidades* program transferred money directly to beneficiary families that complied with the health care plan, including mandatory antenatal care visits in pregnancy and reproductive health talks. Through this study of moderate quality it was shown that there was a long-term effect of the program with longer exposure being associated with more ANC visits and a higher likelihood to chose a physician/nurse than a traditional midwife at delivery.

A similar study of high quality was carried out in Honduras where monetary vouchers were awarded women who kept up-to-date with the routine antenatal and postnatal health services [21]. Authors showed an impact on ANC attendance with an increase of up to 20 percentage points in the catchment area.

It has been stated that demand-side financing is not enough in order to secure good quality services; innovative approaches to health system strengthening are also needed in order to ensure sustainability [22]. This was an underlying concept in efforts to increase maternal health care utilisation for poor women in Cambodia. In this program, a voucher scheme at community level and a health care fund providing free services at hospital level were combined with interventions to improve performance of health service providers through performance-based contracting and delivery incentives [19]. Due to the multiple interventions and the lack of a strong study design the effectiveness of the different components is difficult to assess and distinguish from the general economic and societal development in Cambodia. Ir et al however indicate that institutional delivery rates increased sharply over the two years (from 2006 to 2008) the program was in effect and that the increase was more substantial in districts with the full package compared to districts lacking demand-side interventions [19].

We found only two studies related to contraceptive prevalence rate (MDG 5.3). One study evaluated an intervention using a voucher scheme to improve the rate of modern contraceptive use [23]. This study, which was situated in Nicaragua and of moderate quality, primarily targeted adolescents in disadvantaged areas of Managua. Over 28,000 vouchers were distributed, which were valid for one consultation and one follow-up visit for family planning, pregnancy test, antenatal care, STI treatment or a combination of these. Study results showed that voucher recipients had a significantly higher use of modern contraceptives than non-receivers (48% vs. 33%). Another study, of moderate quality, evaluated the effects of the Mexican *Oportunidades* scheme, described above, on the use of modern contraceptives [24]. Authors found mixed effects of the intervention on contraceptive use. In the early stages of the program the beneficiaries of the program increased their contraceptive use more than the controls, but later on the effect of the program could not exhibit any difference between groups.

We found no studies targeting adolescent birth rates (MDG 5.4) or unmet need for family planning (MDG 5.6).

### Meta-analysis

Despite the heterogeneity of the included studies in terms of intervention and study design we undertook meta-analyses to investigate the pooled effect of study results on health facility delivery and antenatal care rate. Data was extracted based on demonstrated results to fit into a before-after estimate. Test for heterogeneity was performed as part of the R package and T^2^ and I^2^ statistics displayed [11]. It is however important to note that these estimates are often imprecise, especially considering the small number of studies included.

**Figure 4:**
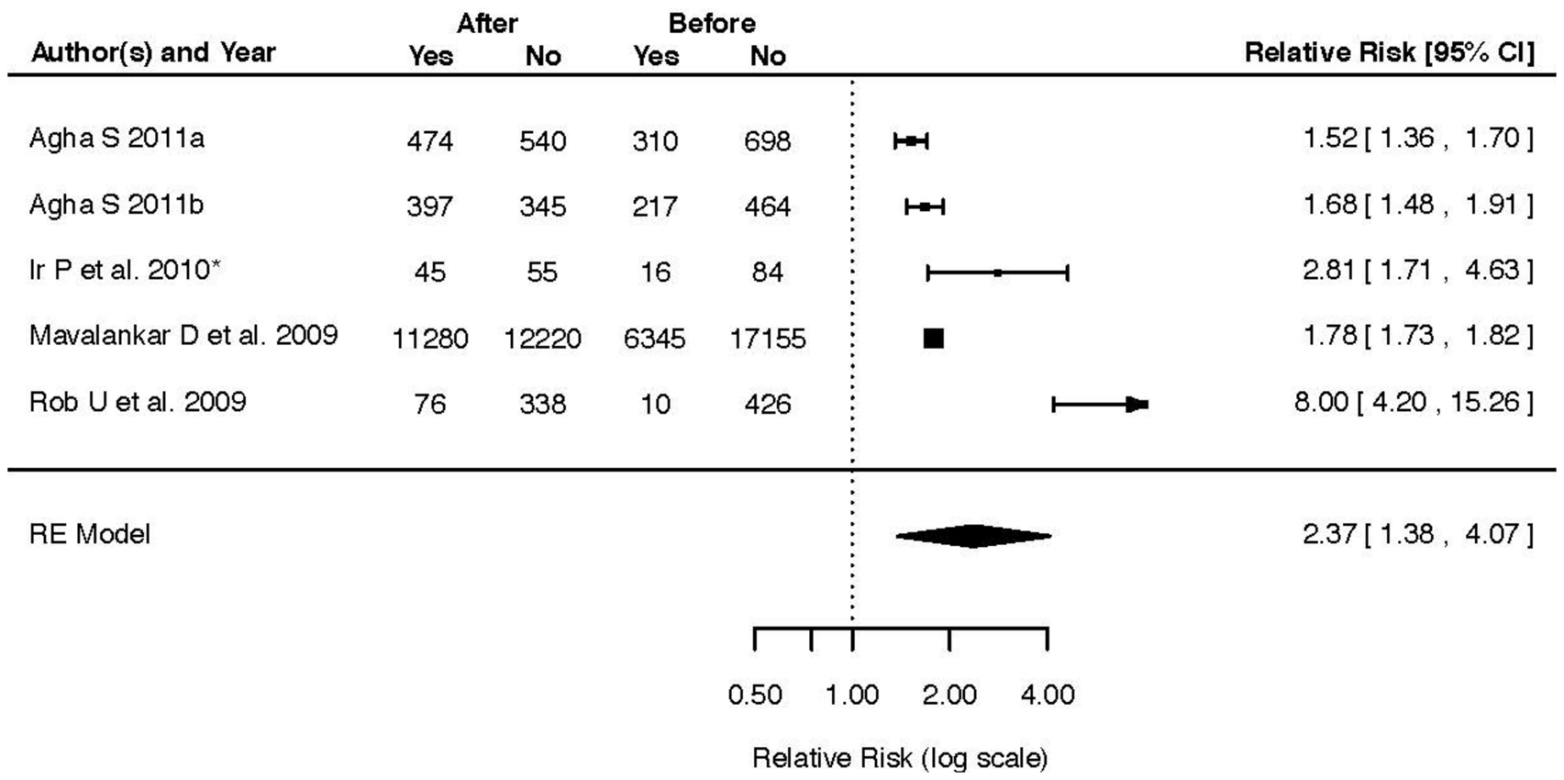
Forest plot of five studies displaying the effect of incentives interventions on health facility delivery rate before and after intervention.

**Figure 5:**
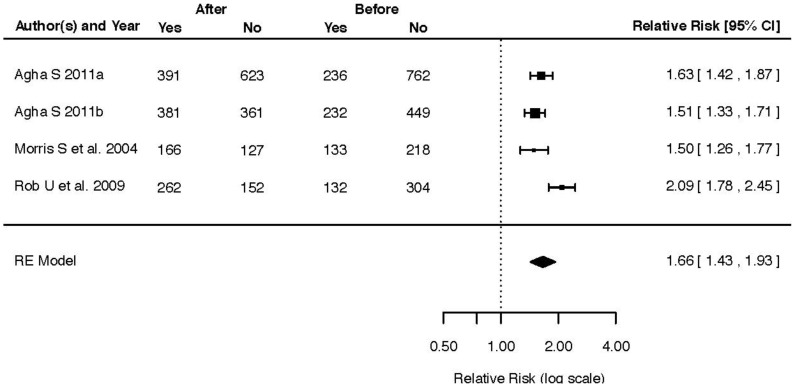
Forest plot of four studies displaying the effect of incentives interventions on antenatal care coverage before and after intervention.

Five studies with a total of 25,625 participants [12], [15]–[17], [19] displaying differences in health facility delivery rate before and after intervention were included and rendered a pooled effect of RR 2.37 (95% CI 1.38–4.07, T^2^ = 0.3487, I^2^ = 98.91%) (Figure 4). Three studies with a total of 2,229 participants compared intervention and control areas for the same outcome [14], [16], [22]. The pooled effect of these studies was RR 3.60 (95% CI 0.94–13.74, T^2^ = 1.3415, I^2^ = 99.34%). The effect of incentives on antenatal care coverage before and after intervention was reported in four studies with a total of 2,476 participants [15]–[17], [21]. The pooled effect on ANC coverage of these studies was RR 1.66 (95% CI 1.43–1.93, T^2^ = 0.0175, I^2^ = 75.97%) (Figure 5). Four studies with a total of 2,522 participants [14], [16], [21], [22] evaluating the same outcome by comparison of intervention and control areas demonstrated a pooled effect of RR 1.67 (95% CI 1.08–2.59, T^2^ = 0.1852, I^2^ = 97.63%).

### Incentives Strategies for MDG4

None of the included studies had under-5 mortality (MDG 4.1) or infant mortality (MDG 4.2) as an outcome variable. However, the already mentioned article by Lim et al evaluating India’s *Janani Suraksha Yojana* program included perinatal and neonatal deaths as mentioned above.

Four studies evaluating different incentive schemes on measles vaccination rates (MDG 4.3) were included, all with a high quality [21], [25]–[27]. Three of the studies were from Latin America. The three studies shared similar interventions, providing eligible families with monetary compensation on a monthly basis as long as the family complied with a preventive health care plan. Two of the studies, in Mexico and Nicaragua could show small but significant effects on measles vaccination rates [26], [27] with an increase of about three percentage points. The study from Honduras was however not able to demonstrate a program effect on measles vaccinations [21].

The only study included that did not use money as an incentive was a study from Bangladesh where a small material incentive was added to an immunisation campaign, rewarding families with lentils and metal plates upon completion of vaccination. Banerjee et al could thus show that this small addition doubled the likelihood of children being fully immunised (RR 2.2, 95% CI 1.5 to 2.8) compared to children participating in an immunisation campaign without material incentives.

### Policy Interventions

Two studies, both of high quality, were found that evaluated targeted policy interventions for improved maternal and child health. In the first study the effects of two health programs in Peru on women’s choice whether to deliver at a health facility with emergency obstetric care (EmOC) capacity or not were evaluated [28]. One of the health programs, *Proyecto 2000*, aimed at increasing quality and utilisation of EmOC facilities in districts reporting the highest maternal and neonatal mortality rates. The other program, *SMI Program*, provided delivery care coverage through a national maternal and child health insurance to the poorest households. Results show that the targeted policy intervention providing the poorest households with free delivery care was more likely to influence the choice of delivery place than the quality enhancing intervention.

The other policy intervention study included for review focused on vaccination coverage where a policy change regarding the distribution of vaccines resulted in increased vaccination coverage in the hard-to-reach areas of Bangladesh [29]. The study team worked together with local health care officials to identify and redesign working patterns with a clearly stated aim to reach geographically difficult areas. This policy change aimed at a disadvantaged population was part of package intervention including training sessions and the development of community support groups. This intervention design makes it difficult to assess the contribution of the changed working patterns but the clearly stated aim to reach geographically remote areas qualifies the study to be included in the present review as a targeted intervention.

### Culturally Adapted Interventions

Only one study was found that reported effects on either MDG4 or MDG5 outcomes by culturally adapted interventions. This study, rated of moderate quality, evaluated a program targeting the custom of packing the umbilical stump with cow dung among the Masai population in Kenya and Tanzania [30]. This cultural habit caused a high number of tetanus related child deaths and through a health promotion program delivered by locally recruited traditional birth attendants, child mortality was sharply reduced and tetanus rates more or less obliterated. The intervention program started in 1981 and the effect was sustained through follow up in 1999. Key components of this program were that it recognised the cultural significance of birthing rituals and negotiated culturally acceptable alternatives while at the same time mobilising local health promoters in order to maintain changed behaviour.

## Discussion

### Principal Findings

We have made a comprehensive collection and assessment of available evidence of the effects of targeted interventions to reduce inequity in maternal and child health. The overall number of studies found was small, and moreover, the approaches to specifically tailored interventions to target disadvantaged populations are limited. During the screening process we identified 1,101 studies reporting on effectiveness of interventions aimed to improve maternal and child health, although only 18 were specifically designed to improve health outcomes for disadvantaged populations. Out of these, a majority (15) evaluated interventions using cash or material incentives, two were aimed at changing policy and only one study was culturally adapted. The almost complete lack of studies that address cultural issues in relation to maternal and child health is alarming. Even if the outcome criteria of this review limited the inclusion there are abundant examples of cultural customs and traditions destructive for women’s and children’s health globally. The fact that almost all of the included studies addressed poverty further re-enforces the notion that inequity in health is mostly considered to be a matter of income and that inequity based on for example ethnicity and culture is neglected.

### Strengths and Weaknesses of the Available Evidence

Most studies evaluating different incentive programs on maternal health used quasi-experimental designs with control groups and only three studies were classified as weak using the six item quality criteria. The diversity of intervention designs and choice of control groups made it difficult however to compare studies. The studies evaluating incentive programs for child health were generally of a higher scientific quality but the outcome measure was mainly measles vaccination rate, which is better suited for randomized controlled trials, considered by many to be the gold standard for research design. The two policy implementation studies included were both rated strong. However, due to the uniqueness of both studies and the low number of studies found it is impossible to draw any inference from the gathered evidence on policy interventions.

We conducted four meta-analyses on the included material focusing on the effect of incentives programs on ANC coverage and health facility delivery rate. The pooled effect of the included studies showed a positive impact of these initiatives on the outcomes. It must however be noted that the included studies had quite different intervention designs, and it might be argued that the analyses are inappropriate because of this. Furthermore, the number of studies in each category is limited and none of the studies had a randomized controlled design. Keeping these shortcomings in mind, we however believe that the results from the meta-analysis add to the overall picture of the benefits of targeted interventions.

### Strengths and Weaknesses of the Review

We have aimed to include both published and unpublished studies and we allowed for a wide range of study design in order to get an as comprehensive picture as possible. However, it might be possible that we still have missed some relevant studies, especially in the light of the low number of studies included. The selection criteria was however rather strict, focusing only on MDG 4 and 5 outcomes. Thus studies measuring DTP vaccine coverage, care-seeking behaviour for childhood illnesses and use of bednets, for example, were excluded despite the variables’ close connection and potentially similar pathways for change. By focusing on the MDG 4 and 5 outcomes we however kept the research question of the review within the framework of key indicators agreed upon by the global community to measure maternal and child health outcomes and manage to show the need for further follow-up of these indicators.

Another methodological concern is the question of what is a targeted intervention. Many studies claimed to target a disadvantaged population, such as slum dwellers or rural populations. But to extend general health interventions to disadvantaged population groups is not the same as addressing the underlying determinants causing the population to be disadvantaged. To some extent it can be argued that the lack of health care provision is a driving force for inequity and by providing universal coverage the health inequities will be reduced. This is however a simplification of the equity problem and only by acknowledging the structural complexity is it possible to adequately address the unjust differences in health outcomes. Therefore we have chosen to focus this review on interventions that were tailored to specifically overcome structural obstacles to an equitable health distribution.

### Implications for Policy and Future Research

From the evidence compiled in this review it can be concluded that incentive programs, if designed in an appropriate manner, might be a possible way to reduce inequities in maternal and child health care provision and ultimately death. Our results are in line with previous reviews on cash transfer schemes [5], [31]. It might however be noted that the design and context might play important roles for the success or failure when scaling up incentives programs [25].

This review has exposed a major research gap. The potential interventions targeted at disadvantaged populations addressing specific structural determinants of health are likely to be numerous. Yet there is a lack of research reports evaluating such efforts. If this is due to a general lack of tailored interventions or a lack of interest from the research community to explore this strategy to reduce inequity is difficult to say. To meet the public health needs in the years to come, when equity in health is surfacing as one of the main challenges to the global health community there is not only a need for universal health coverage. Scaling up targeted interventions addressing specific structural determinants of inequity in health is a necessity unless we are to continue to depend on the “trickle down” philosophy so far known to benefit the better off to a larger extent [32]–[34]. Scaling up, however, will require implementation research, focusing on measurable outcomes and transparent process descriptions and evaluations.

## Supporting Information

Figure S1 **PRISMA flow diagram.**
(PDF)

Appendix 1 **Search strings**
(PDF)

Checklist S1 **PRISMA checklist.**
(DOC)
