# Supplementary material for: Targeted Interventions for Improved Equity in Maternal and Child Health in Low- and Middle-Income Settings: A Systematic Review and Meta-Analysis
Source: PLoS One. 2013 Jun 20;8(6):e66453. doi: 10.1371/journal.pone.0066453 (PMC3688766; doi:10.1371/journal.pone.0066453)
Supplement: Figure S1 — PRISMA flow diagram. (PDF) [file pone.0066453.s001.pdf]

PRISMA 2009 Flow Diagram

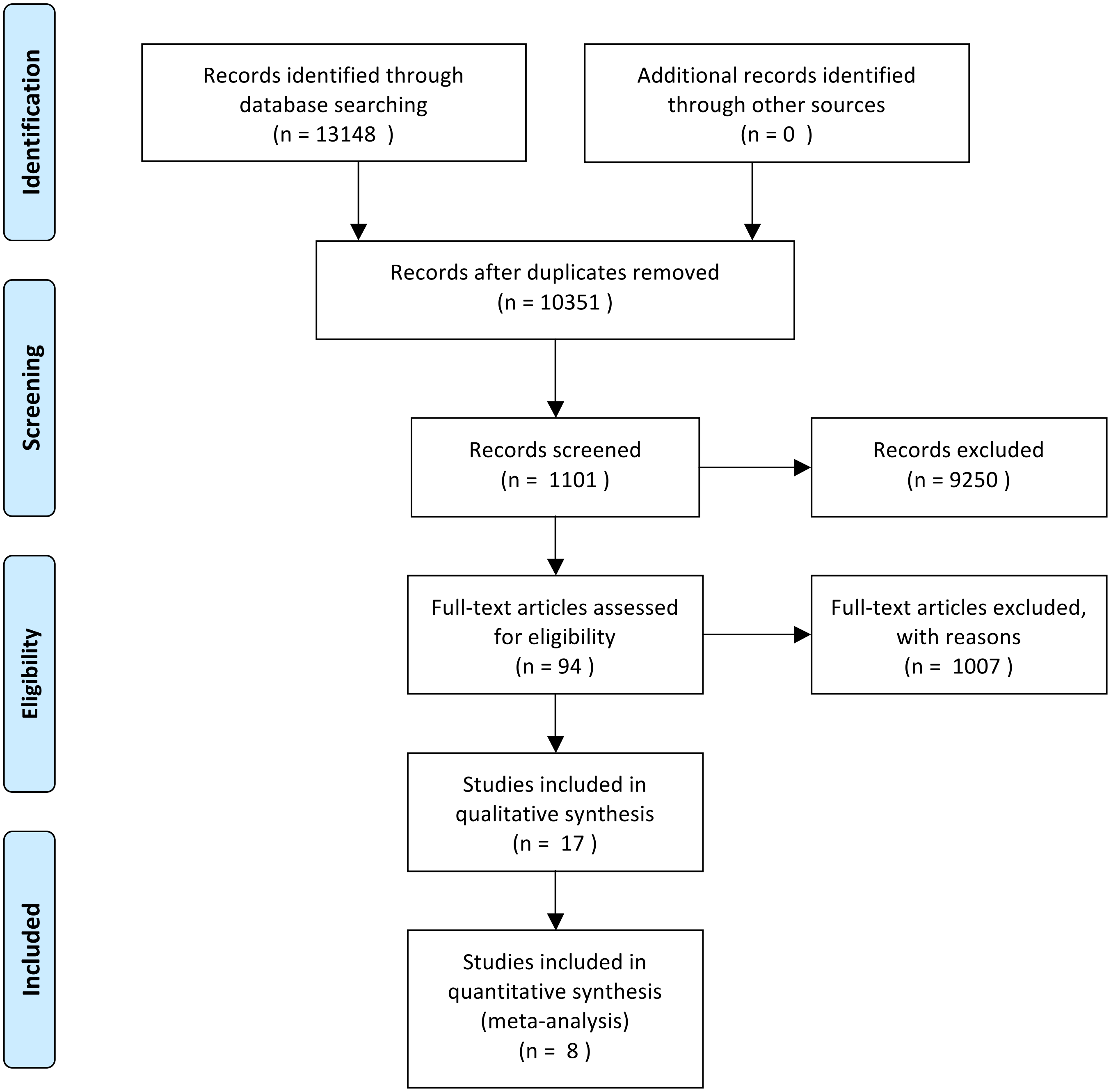

From: Moher D, Liberati A, Tetzlaff J, Altman DG, The PRISMA Group (2009). *P*referred *R*eporting *I*tems for *S*ystematic Reviews and *M*eta-*A*nalyses: The PRISMA Statement. PLoS Med 6(6): e1000097. doi:10.1371/journal.pmed1000097

For more information, visit www.prisma-statement.org.
